# Supplementary material for: Early to Late VSV-G Expression in AcMNPV BV Enhances Transduction in Mammalian Cells but Does Not Affect Virion Yield in Insect Cells
Source: Vaccines (Basel). 2025 Jun 26;13(7):693. doi: 10.3390/vaccines13070693 (PMC12297889; doi:10.3390/vaccines13070693)
Supplement: Supplementary file 1 [file vaccines-13-00693-s001.zip › vaccines-3668095-supplementary.pdf]

Supplementary material

A.

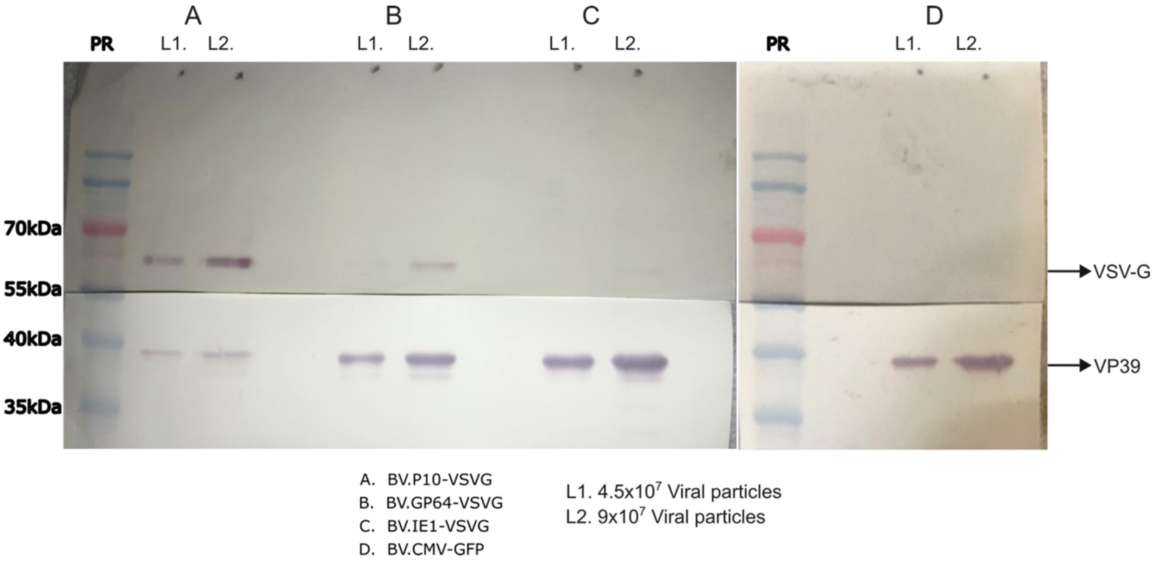

B.

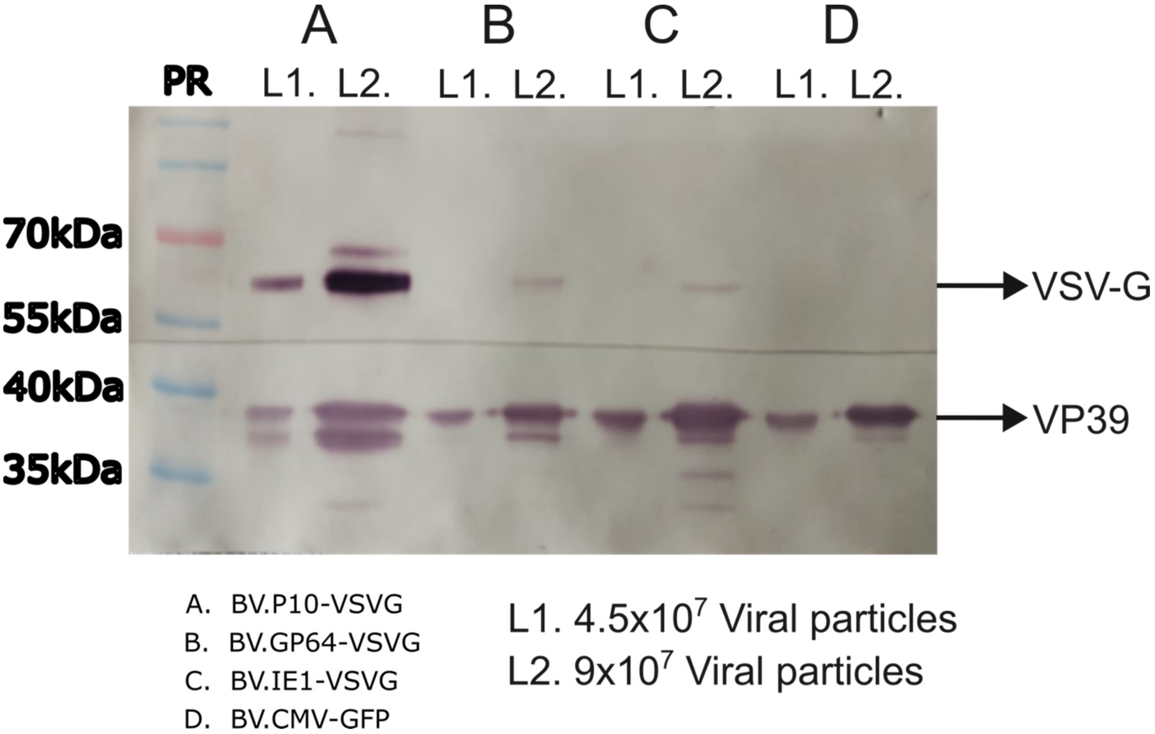

Figure S1. Immunodetection of VSV-G in recombinant AcMNPV. Western blot assays where ultraconcentrated samples of recombinant BVs were analyzed with anti-VSV-G and anti-VP39 antibodies. At the top of each lane (with references at the bottom) the identity of the samples studied are indicated. The most important protein sizes of molecular weight bands are indicated.
